# Single-Cell Transcriptomic Analysis Reveals Mitochondrial Dynamics in Oocytes of Patients With Polycystic Ovary Syndrome

**DOI:** 10.3389/fgene.2020.00396

**Published:** 2020-04-30

**Authors:** Lingbin Qi, Boxuan Liu, Xian Chen, Qiwei Liu, Wanqiong Li, Bo Lv, Xiaoyu Xu, Lu Wang, Qiao Zeng, Jinfeng Xue, Zhigang Xue

**Affiliations:** ^1^Department of Regenerative Medicine, Tongji University School of Medicine, Shanghai, China; ^2^Precision Medicine Center, The Second People’s Hospital of Huaihua, Huaihua, China; ^3^Department of Gynecological Minimal Invasive Center, Beijing Obstetrics and Gynecology Hospital, Capital Medical University, Beijing, China; ^4^Reproductive Medicine Center, Tongji Hospital, Tongji University, Shanghai, China; ^5^Center of Reproductive Medicine of Ji’an Maternal and Child Health Hospital, Ji’an, China

**Keywords:** polycystic ovary syndrome, mitochondrial dynamics, oocyte, single-cell sequence, transcriptomic analysis

## Abstract

Polycystic ovary syndrome (PCOS), characterized by polycystic ovarian morphology, ovarian follicular maturation arrest, and hormonal disorders, affects numerous women in the reproductive age worldwide. A recent study has found that mitochondria are likely to play an essential role in oocyte quality. However, it is still unclear whether oocyte development failure is associated with mitochondria in patients with PCOS. We analyzed the single-cell RNA sequencing data from the previous study, including data from 14 oocytes from 7 healthy fertile women and 20 oocytes from 9 patients with PCOS at the germinal vesicle (GV) stage, metaphase I (MI) stage, and metaphase II (MII) stage. We revealed the transcriptomic dynamics by weighted gene co-expression network analysis (WGCNA) and investigated the differences between stages using PCA and Deseq2 analyses to identify the differential expression genes (DEGs). Gene ontology (GO) was performed using clusterProfiler R package and Metascape. Our results indicated that specific gene modules were related to different stages of oocyte development using WGCNA. Functional enrichment analysis and gene co-expression network analysis found significant enrichment of the mitochondrial regulation genes at the GV stage. PCA (principal component analysis) and differential gene expression analysis suggested that GV was significantly different from the MI and MII stages between the two groups. Further analysis demonstrated that the upregulated differentially expressed genes at the GV stage of patients with PCOS mainly related to mitochondrial function, such as *COX6B1, COX8A, COX4l1*, and *NDUFB9*. Meanwhile, these genes tended to be activated at the MII stage in healthy cells, suggesting that some mitochondrial functions may be prematurely activated at the GV stage of PCOS oocytes, whereas this process occurs at the MII stage in healthy oocytes. Collectively, our study showed that aberrant mitochondrial function at the GV stage may contribute to a decline in oocyte quality of PCOS patients.

## Introduction

Polycystic ovary syndrome (PCOS) is a common disorder affecting 5–20% of the women of reproductive age worldwide. PCOS is mainly characterized by hyperandrogenism, ovulatory dysfunction, and polycystic ovarian morphology (PCOM) (Azziz et al., 2016). The interaction between insulin resistance (Dunaif, 2006), hyperinsulinemia (Landay et al., 2009), and gonadotropin disturbance (Hsu et al., 2009) can induce PCOS, but the pathogenesis is still unclear. Currently, the treatment strategies for PCOS mainly include medical treatment, ovarian surgery, and *in vitro* fertilization (IVF) therapy (Child et al., 2001; Harborne et al., 2003; Hendriks et al., 2007; Requena et al., 2008; Witchel et al., 2019). Women diagnosed with PCOS, who undergo IVF treatment, show a high probability for pregnancy and reach the mean level of the pregnancy rate (Nahuis et al., 2011). However, several serious problems exist. Besides the high risk of ovarian hyperstimulation syndrome and preeclampsia, oocyte amounts and quality obtained from patients with PCOS undergoing IVF are worse than those from healthy fertile women (Fauser et al., 2004; Ahmadi et al., 2013).

Mitochondria are the center of energy metabolism, and can both supply enough energy for cells and regulate cellular survival and development (Chan, 2006; Martinou and Youle, 2011; Spinelli and Haigis, 2018). Accumulating evidence has shown that mitochondria play an essential role in oocyte maturation, influencing oocyte and subsequent embryonic development (Babayev and Seli, 2015; May-Panloup et al., 2016). Mitochondrial dysfunction affects oocyte maturation, which can consequently decrease female fertility (Harvey, 2019). It can be inferred that a decline in oocyte quality in patients with PCOS may be related to abnormal mitochondrial function. However, the intracellular alterations and mitochondria-associated mechanism in oocytes of PCOS are still unclear owing to the limitations in patient material and technologies.

In this context, we analyzed the single-cell RNA-seq (scRNA-seq) data of healthy and PCOS oocytes from different stages of its maturation, including germinal vesicle (GV), metaphase I (MI), and metaphase II (MII). Combined with the bioinformatics algorithms, we determined the mitochondrial dynamics in PCOS oocytes and explored whether aberrant mitochondrial function is associated with the failure of oocyte development in patients with PCOS.

## Materials and Methods

### Data Retrieval

The scRNA-seq data for analyses were obtained from parts of a previous study published by our group, inclusion criteria and ethical statements have been previously described (Liu et al., 2016). Briefly, ovarian stimulation and oocyte retrieval were performed according to the published protocols (Fauser et al., 2004). Each picked up oocyte was transferred to a tube containing lysis buffer and stored at −80°C for further analysis. We conducted single-cell RNA-sequencing followed by the published Smart-seq2 protocol (Picelli et al., 2013) and sequence based on illumina HiSeq 2500.

**FIGURE 1:**
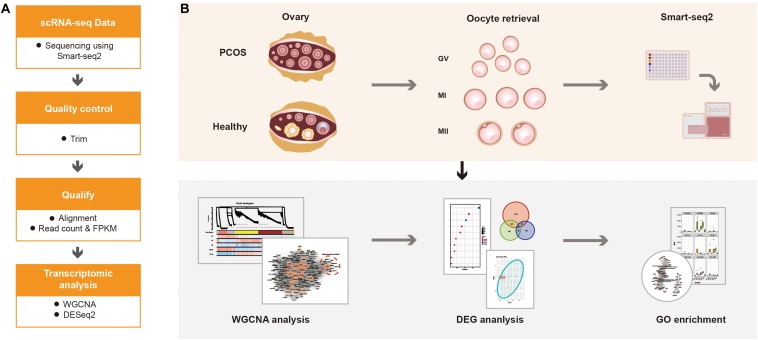
The schematics of the study design. **(A)** The progress of data alignment. After acquiring the raw data of Smart-seq2, the adaptor contaminants and low-quality reads were trimmed. Then, number of mapped read in the BAM file of each sample were aligned to read count and FPKM. **(B)** The progress of the study. scRNA-seq data were obtained at different oocyte development stages (GV, MI, and MII) from healthy women and patients with PCOS, the processed data were used for WGCNA, differential gene expression analysis and GO enrichment analysis to detect the dynamic changes of transcriptome.

For calculating the expression of genes, the adaptor contaminants and low-quality based on the scRNA-seq reads were trimmed by Trimmomatic (V0.33) (Bolger et al., 2014), then the clean reads were aligned to hg38 reference genome using STAR (version 2.7.1a) (Dobin et al., 2013). The number of mapped reads in the BAM file of each sample was counted, then the gene expression was defined using read count (Figure 1A). Additionally, normalized counts Fragments Per Kilobase Million (FPKM) was calculated based on the previous paper (Trapnell et al., 2014). According to the protocol, the read count was used for DESeq2 to calculated the differentially expressed genes (DEGs), FPKM was used for WGCNA analysis (Figure 1B).

### Weighted Gene Co-Expression Network Analysis (WGCNA)

To investigate the gene expression network during the maturation process of oocytes, we performed WGCNA (V1.68) (Langfelder and Horvath, 2008) and determined the relationship of gene co-expression in different stages between healthy individuals and individuals with PCOS. The criteria of filtering were clustering height above 15000 or below 10. The power used in WGCNA was set according to the SoftThreshold, and we plotted module detection via dynamic tree cutting. For the demonstration of the relationship between different modules, results were visualized using module and eigengene relation heatmap, and the gene co-expression network was extracted and further processed using MCODE in Cytoscape software (V3.7.2) (Shannon et al., 2003). For a distinct and precise representation of the data, genes contained in the significantly relevant modules were extracted and analyzed using Metascape (Zhou et al., 2019).

### Gene Clustering and Differential Expression Analysis

To explore the clusters in different stages, we used the principal component analysis (PCA) and visualized the dotplots by selecting the top two principal components. Differential gene expression analysis was performed using the DESeq2 package (V1.24.0) (Love et al., 2014). Briefly, read counts were input to build dds objects, and DEGs were extracted from the dds results. DEGs at different stages were illustrated using a volcano plot according to the EnhancedVolcano package (V1.2.0) (Blighe et al., 2019). Genes with a *p*-value < 0.05 and absolute fold change ≥2 were regarded as differentially expressed genes.

### Gene Ontology (GO) and KEGG analysis

To detect the genome-wide expression profiles between healthy and PCOS oocyte, we performed gene set enrichment analysis (GSEA) for hallmark gene sets and GO gene sets separately (Mootha et al., 2003; Subramanian et al., 2005) using datasets of whole samples in these groups. Gene ontology enrichment analysis was conducted using the clusterProfiler package (V3.12.0) (Yu et al., 2012). Before analysis, we transferred gene names from “symbol” to “entrezid” according to org.Hs.eg.db package (V3.8.2) (Marc, 2019). We harvested genes using biological progress (BP) subontology. Terms with the *p*-value < 0.05 were considered and enriched as significant. Dotplot and gene-concept networks were illustrated using the enrichplot package (V1.4.0) (Yu, 2019). Relative expression of selected genes involved in GO terms was calculated using samples filtered by clustering height above 15000 or below 10 and Boxplots were illustrated using the ggplot2 package (V3.2.1) (Ginestet, 2011). Genes involved in particular GO sets were highlighted in the KEGG pathway using clusterProfiler package (V3.12.0) (Yu et al., 2012).

### Statistical Analysis

Statistical analyses in Figure 5 were performed by GraphPad Prism (V8.2.1). The FPKM data of selected mitochondria-related genes were used for testing by unpaired, two tailed Mann-Whitney *U* tests. *p*-value < 0.05 were regarded as statistically significant and marked with asterisk.

## Results

### WGCNA Reveals Different Genetic Dynamics of Maturation Between Healthy and PCOS Oocytes

**FIGURE 2:**
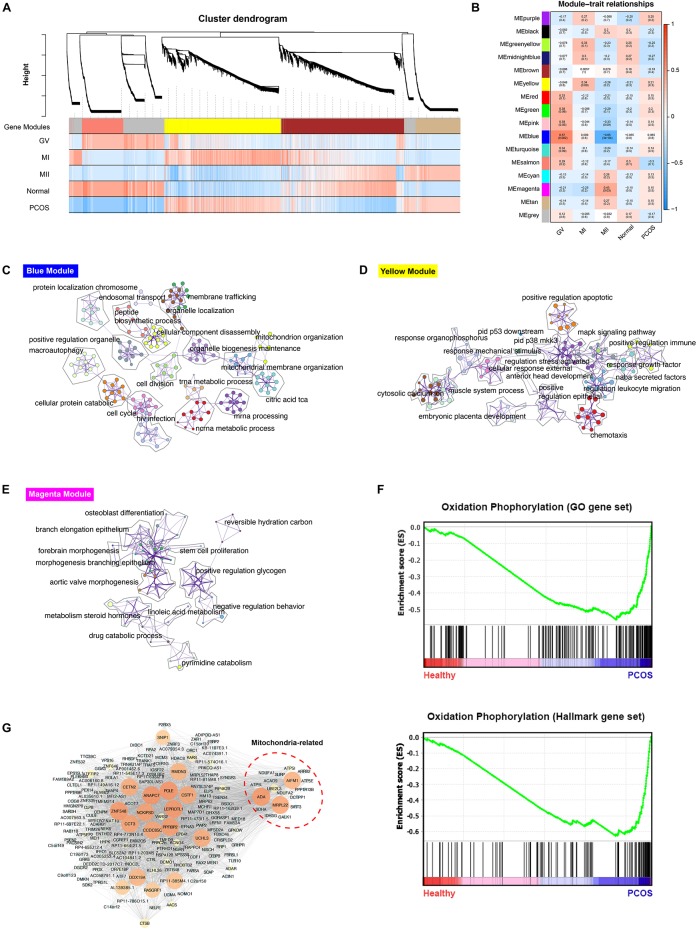
WGCNA analysis of healthy and PCOS oocytes in different stages. **(A)** The dendrogram of gene modules built by WGCNA. Bars represent the correlation between genes in different developmental stage of oocyte and gene modules. Red means positively correlation and up-regulated at this stage; blue means negatively correlation and down-regulated at this stage. **(B)** Module-trait relationship between different gene modules. Number in each cell represent the degree of correlation, and red means positively correlation at this stage; blue means negatively correlation at this stage. Different colors represent the diverse specific gene modules detected by WGCNA. C-E. Network of enriched GO terms of genes containing in blue module **(C)**, yellow module **(D)** and magenta module **(E)** separately, colored by GO cluster ID. **(F)** The results of Gene set analysis (GESA) for hallmark gene sets (up) and GO biological processes gene set (down). The enrichment score (ES) means value of maximum deviation from 0 of the running sums and each line on the button represent the different genes of gene set. **(G)** Co-expression network of hub genes containing in blue module illustrated by the Cytoscape. The large size and the bright color of nodes represented high MCODE score of genes.

The scRNA-seq data of 6 GV oocytes, 5 MI oocytes, 3 MII oocytes from 7 healthy fertile women and 7 GV oocytes, 4 MI oocytes, 9 MII oocytes from 9 PCOS patients were analyzed. The clinical variables of these 16 donors confirmed the phenotype of PCOS patients, including significant decreased of antral follicle, LH, LH/FSH, testosterone, sex hormone-binding globulin (SHBG) and ovarian volume (*p*-value < 0.05) (Table 1). For exploring the genetic program macroscopically, we profiled 63129 genes from these 35 samples using WGCNA and 16 gene co-expression modules were identified (Figure 2A). Particularly, we found that the blue module was the most significant and correlated with the GV stage, while the yellow module was the most significant and correlated with the MI stage, and magenta module was the most significant and correlated with the MII stage (Figure 2B). GO functional enrichment of hub genes in these three modules were analyzed using Metascape (Zhou et al., 2019). In the blue module, genes were mainly involved in the ncRNA metabolic process, mitochondrion organization and others, while chemotaxis, pid p53 downstream pathway and others were abundant in the yellow module, and reversible hydration of carbon dioxide, cell-cell adhesion and others were highly represented in the magenta module (Figures 2C–E, Supplementary Figure S1, and Supplementary Table S1).

**TABLE 1:** The clinical variables of healthy donors and PCSO patients.

	Healthy donors (*n* = 7)	PCOS patients (*n* = 9)	*p*-value
Age (years)	29.7 ± 0.6	27.4 ± 1.1	0.1266
BMI (Kg/m^2^)	18.6 ± 0.4	19.7 ± 0.6	0.1427
SBP (mmHg)	119.2 ± 3.9	116.3 ± 4.4	0.6515
DBP (mmHg)	77.1 ± 2.5	76.4 ± 3.3	0.8739
AFC (antral follicle)	10.1 ± 1.4	>24	<0.001(0.000)**
FSH (mlU/ml)	6.1 ± 0.5	5.7 ± 0.5	0.5516
LH (mIU/ml)	5.0 ± 0.6	8.7 ± 0.6	<0.001(0.000)**
LH/FSH	3.9 ± 0.3	6.2 ± 0.4	<0.001(0.000)**
Insulin (IU)	10.5 ± 1.9	12.7 ± 2.1	0.464
Testosterone (nmol/L)	1.1 ± 0.1	1.6 ± 0.2	<0.05(0.0311)*
Androstenedione	2.2 ± 0.3	2.3 ± 0.2	0.7234
Sex Hormone-Binding Globulin (SHBG, nmol/L)	38.0 ± 2.5	53.7 ± 5.4	<0.05(0.0313)*
Ovarian volume total	3809.6 × 408.0	5789.8*555.2	<0.05(0.0165)*

To find the biological pathways which play a key role during the dynamic progression of oocyte development, the gene set enrichment analysis (GESA) was performed and oxidative phosphorylation was significantly enriched in PCOS group using both hallmark gene sets and GO gene sets, suggesting the significant difference in mitochondrial function between healthy and PCOS oocytes (Figure 2F). Furthermore, only hub genes in blue module which related to GV stage enriched with mitochondrial function terms (Figure 2C). Therefore, we further analyzed the genes in co-expression network of blue modules using Cytoscape MCODE algorithm. The hub genes with high MCODE score were related to many important functions during the meiosis and oocyte maturation, including regulation of microtubule and centrosome (*RMDN3, CETN2*) (Salisbury et al., 2002; Gomez-Suaga et al., 2017), DNA repair and replication (*POLE*) (Frigola et al., 2017), oocyte maturation and cleavage (*ANAPC7, CSTF1*) (Elkon et al., 2013; Wild et al., 2018), nucleic acid binding (*ZNF548, DDX19A)* (Li et al., 2015; Nasri et al., 2019). Notably, a subset of genes related to mitochondrial functions was identified, including *MRPL22, AIFM1, ADA, ATP5L*, *NDUFA1, NDUFA2* which are broadly involved in nucleoside triphosphate metabolic processes, mitochondrial oxidation respiratory and energy generation (Figure 2G). Overall, these results suggested that mitochondrial function may have an essential impact in GV stage progression.

### Significant Difference in Transcriptome Variations Between Healthy and PCOS Oocytes at the GV Stage

**FIGURE 3:**
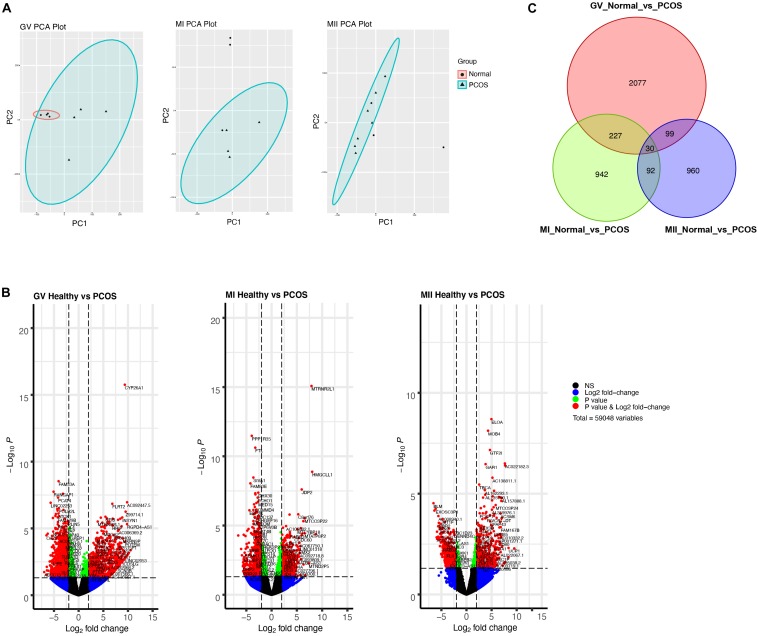
Single-cell RNA-seq transcriptome profiling of healthy and PCOS oocytes. **(A)** PCA plot of single-cell transcriptomes at GV **(left)**, MI **(middle),** and MII **(right)** stage. **(B)** Volcano plot shows significant DEGs of oocytes in GV **(left)**, MI **(middle)**, MII **(right)** stage between healthy and PCOS group. **(C)** Venn diagram shows the overlap of the DEGs in different stages.

To further explore the discrepancy between healthy and PCOS oocytes in the GV, MI, and MII stages, we clustered genes using PCA. We identified that the clusters were markedly different in GV oocytes between the healthy and PCOS groups, while similar clusters in most of the oocytes at the MI and MII stages between the two groups were not observed (Figure 3A). We collected the highly-differential genes at diverse stages from healthy and PCOS oocytes and visualized the data by volcano plots (Figure 3B and Supplementary Table S2). By setting the standard of *p*-value < 0.05 and fold change ≥2, a total of 2426, 1298, and 1181 DEGs were identified in the GV, MI, and MII stages, respectively. Prominent DEGs were identified in each stage, for instance, the downregulation of *CYP26A1, MTRNR2L1, ELOA* and upregulation of *FAM53A, PPP1R35, BLM* at GV, MI, MII stages in PCOS oocytes, respectively (Figure 3B). It has been reported that *CYP26A1, MTRNR2L1, ELOA*, and *FAM53A* were all related with ovarian carcinomas, and *CYP26A1* may acts as a meiosis-inhibiting factor and related with the gonadal retinoic acid-degradation (Rodriguez-Mari et al., 2013). In addition, DEGs from different stages showed more independence and less overlap between each other (Figure 3C and Supplementary Table S3). These results suggest that different development stages are relatively isolated on the transcriptome level, and variations in the GV stage are the most significant.

### An Aberrant Status of Mitochondrial Energy Metabolism in PCOS Iocytes

**FIGURE 4:**
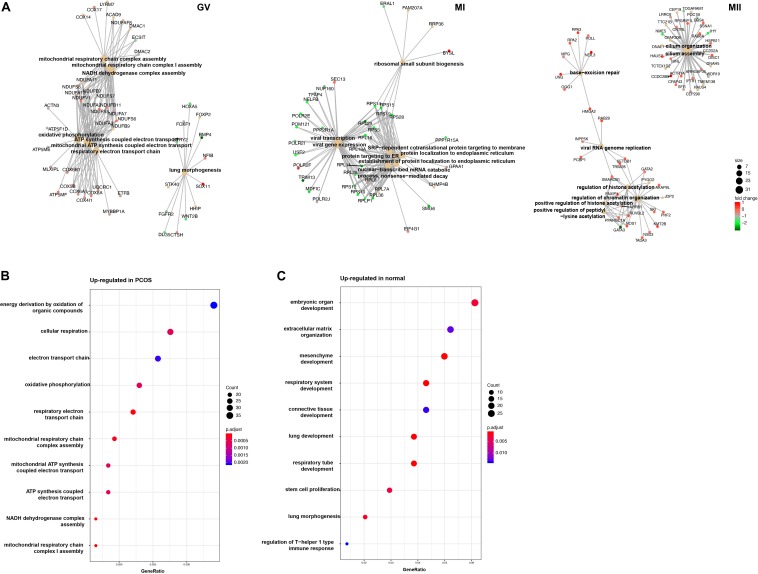
GO analysis for DEGs in different stage. **(A)** Gene connect plot shows the high differential biology progress in GV **(left)**, MI **(middle)**, and MII **(right)** stage between healthy and PCOS oocytes. The node size represented count of genes enriched in GO terms, color represented the fold change between groups. **(B)** Gene ontology of up-regulated genes in PCOS oocytes at GV stage. **(C)** Gene ontology of down-regulated genes in PCOS oocytes at GV stage. The dot size represented the count of genes enriched in GO terms; color represented the *p*-value adjust of terms.

To further investigate the mechanism of differences between healthy and PCOS oocytes at the GV stage, we performed GO analysis using DEGs identified by DEseq2 and found the most of significant enrichment functions of DEGs at the GV stage were mitochondria-related processes, such as ATP synthesis coupled electron transport, mitochondrial respiratory chain complex assembly and similar, however, these terms were not present at the MI and MII stages (Figure 4A). Using GO enrichment analysis, we found that upregulated genes in GV oocytes of patients with PCOS were highly related to mitochondrial energy metabolism (Figure 4B), while upregulated genes in the healthy samples were relevant to oocyte development (Figure 4C), suggesting the mitochondrial function abnormality is likely to be an important reason which lead to the difference between PCOS and healthy oocytes at GV stage.

**FIGURE 5:**
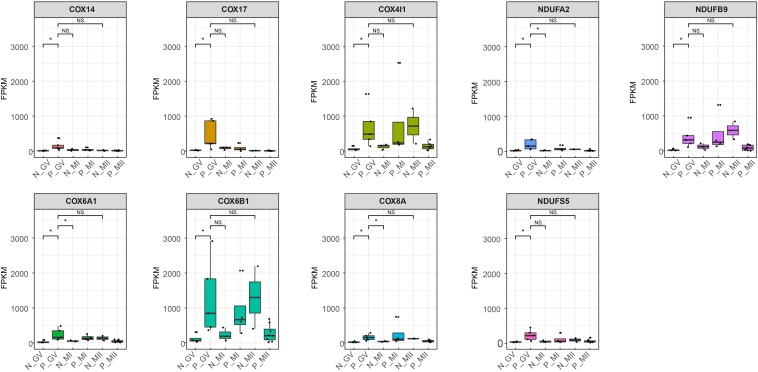
Boxplot shows the dynamics of 9 high differential mitochondria-related genes at different stages between healthy and PCOS oocytes. The FPKM value represent the expression of genes. The line in the box means the media of available samples and *p*-value < 0.05 was regarded as statistical significance and marked with the asterisk (*). Dots represented the oocytes used in each group. FPKM: fragments per kilobase per million.

**FIGURE 6:**
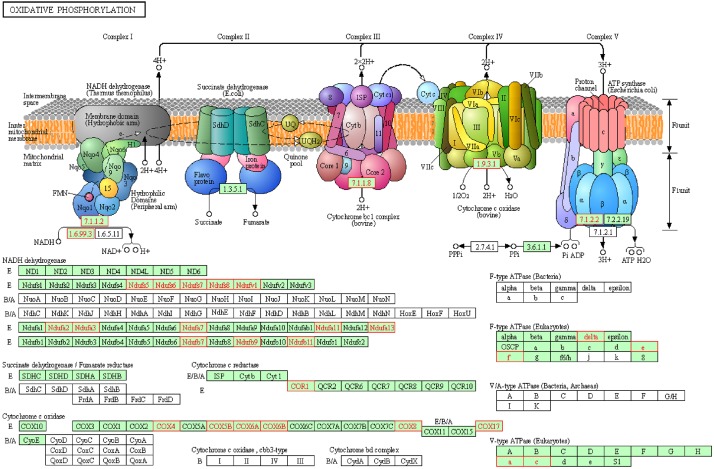
Representative oxidative phosphorylation pathways constructed by KEGG. Red genes represent up-regulated in PCOS oocytes at GV stage.

To reveal the dynamics of mitochondria-related genes at different stages, we compared genes which related to mitochondrial function and were variably expressed at the GV stage between healthy and PCOS oocytes using boxplot (Figures 4A, 5). We found that genes such as *COX6B1, COX8A, COX4l1*, and *NDUFB9* that were highly expressed at the GV stage in PCOS oocytes tended to be activated at the MII stage in healthy oocytes (Figure 5). To investigate the specific function of these genes in mitochondria, we further performed KEGG pathway analysis and found that these genes are involved in most parts of the oxidative phosphorylation process (Figure 6). Based on these findings, we suggest that some mitochondrial functions may be prematurely activated at the GV stage of PCOS oocytes, whereas this process occurs at the MII stage in healthy oocytes.

## Discussion

Previous single-cell transcriptome study of PCOS oocyte reported by Liu et al. (2016) showed genes related to oocyte meiosis and maturation were differentially downregulated and DNA repair-related genes were upregulated at early stages and these DEGs were the potential reasons caused PCOS oocytes disorder. In the present study, we compared the transcriptomic profiles between healthy and PCOS oocytes and found one of the main differences during oocyte development was mitochondrial function, such as upregulated expression of oxidative phosphorylation related genes using GESA analysis. Through detected specific gene modules and dynamic characteristics of co-expression genes using WGCNA, the mitochondria-related genes were identified as hub genes in the gene module related to GV stage. These results were also confirmed using PCA and differential gene expression analysis, the transcriptomic profile was significantly different at GV stage suggesting that the DEGs at the GV stage are vital during the oocyte development process between women with PCOS and healthy women, and these DEGs are mainly related to mitochondrial functions. Additionally, our results also suggested that mitochondrial function, especially the oxidative phosphorylation process, may be prematurely activated at the GV stage in PCOS oocytes, which differ from the healthy oocytes which activated at MII stage. Overall, we give further insight into the mitochondrial dynamics during oocyte maturation and we find that mitochondrial aberrant activation at GV stage may be one of the important factors contributing to reduced oocyte quality in patients with PCOS.

Polycystic ovary syndrome is one of the most common and severe diseases affecting the female reproductive health, a decline in oocyte quality is more susceptive to occur in PCOS patients, and so far, the pathogenesis of PCOS is still unclear (McCartney and Marshall, 2016). The assisted-reproductive technology (ART) presents a way of rescue for PCOS patients, present treatment for PCOS patients includes ovarian stimulation for receiving MII oocyte with subsequent fertilization *in vitro* (Tang et al., 2006; Shalom-Paz et al., 2012), nevertheless, the mature follicular obtained through ovarian stimulation will less than that in women without PCOS for unknown reasons (Liu et al., 2016). The process from GV to MII stages is a key pathway for oocyte development. Although gene abundance have no statistical difference among diverse stages (Wang et al., 2020), dynamic biological process such as differential expression of mitochondria-related genes can be observed. Moreover, the number of mitochondria increases to more than 100,000 in MII oocytes, which is a 1000-fold higher than that in the GV oocytes (Jansen and de Boer, 1998; Darbandi et al., 2017). Thus, the state of mitochondrial function along with cell death and proliferation can also influence oocyte maturation. As the center of energy metabolism and cell apoptosis in most cell types (Vakifahmetoglu-Norberg et al., 2017; Spinelli and Haigis, 2018), mitochondria disorder leads to the failure of human oocyte development (Kristensen et al., 2017; Chiaratti et al., 2018).

In this study, we systematically analyzed the changes in the oocytes of patients with PCOS and healthy fertile women at different developmental stages using single-cell transcriptome sequencing. We found that some genes related to gonadal and oocyte development, such as *CYP26A1* which is upregulated in oocytes during prophase-I meiotic arrest and downregulate when meiosis resumed (Rodriguez-Mari et al., 2013), was downregulated in PCOS oocytes at GV stage. Moreover, We revealed the transcriptomic dynamics during oocyte maturation and found that the mitochondrial function may be prematurely activated since the highly expressed genes at the GV stage of PCOS oocytes show a close relationship with mitochondrial function such as mitochondrial energy metabolism, oxidative phosphorylation process and similar, which differ from the healthy oocytes that these genes were activated at MII stage. It was found that the function of mitochondria is quiescent during follicular development, and energy is mainly provided by the surrounding granular cells (May-Panloup et al., 2016). The premature activation of mitochondria likely leads to the production of harmful metabolites, such as excessive oxygen free radicals, which could induce irreversible damage to the maturating oocyte further resulting in the oocyte retardation (Van Blerkom, 2004; Wang et al., 2009).

However, it should be noted that there are some limitations in this study. Since the women with PCOS often have ovulation disorders which mainly cause infertility (Mahan, 2008), it’s difficult to obtain oocytes from them. Therefore, the number of oocyte samples used in this study is relatively smaller than other oocyte gene expression researches (Li et al., 2019). In addition, the differential expression of mitochondria-related genes presented in this study was quantified by sequencing and data analysis. Further independent validations such as quantitative RT-PCR and functional researches are needed to comprehensively understand the role of the mitochondrial function in the progress of oocyte maturation.

In conclusion, our findings highlight that the transcriptomic dynamics of PCOS oocytes and aberrant mitochondrial function at the GV stage of oocyte development in patients with PCOS were observed. These results deliver novel meaningful insights into the oocyte quality decline in patients with PCOS.

## Data Availability Statement

The data is available to access in GEO SRA and the accession number is PRJNA600740.

## Author Contributions

LQ, BxL, XC, and JX analyzed the data and wrote the manuscript. LQ, BL, QL, and JX performed bioinformatics analysis. WL, BxL, QL, XX, and LW performed the experiments. LQ, ZX, JX, and QZ designed the experiments and revised the manuscript.

## Conflict of Interest

The authors declare that the research was conducted in the absence of any commercial or financial relationships that could be construed as a potential conflict of interest.

## Supplementary Material

FIGURE S1 GO analysis of hub genes in different modules. **(A)** GO terms of hub genes in blue module. **(B)** GO terms of hub genes in yellow modules. **(C)** GO terms of hub genes in magenta module.

TABLE S1 Genes list for three significantly gene modules.

TABLE S2 Differentially expressed genes of GV, MI and MII stages between PCOS and healthy group.

TABLE S3 Overlap genes of venn diagram.
