# Supplementary figures and images for: Single-Cell Transcriptomic Analysis Reveals Mitochondrial Dynamics in Oocytes of Patients With Polycystic Ovary Syndrome
Source: Front Genet. 2020 Apr 30;11:396. doi: 10.3389/fgene.2020.00396 (PMC7203476; doi:10.3389/fgene.2020.00396)

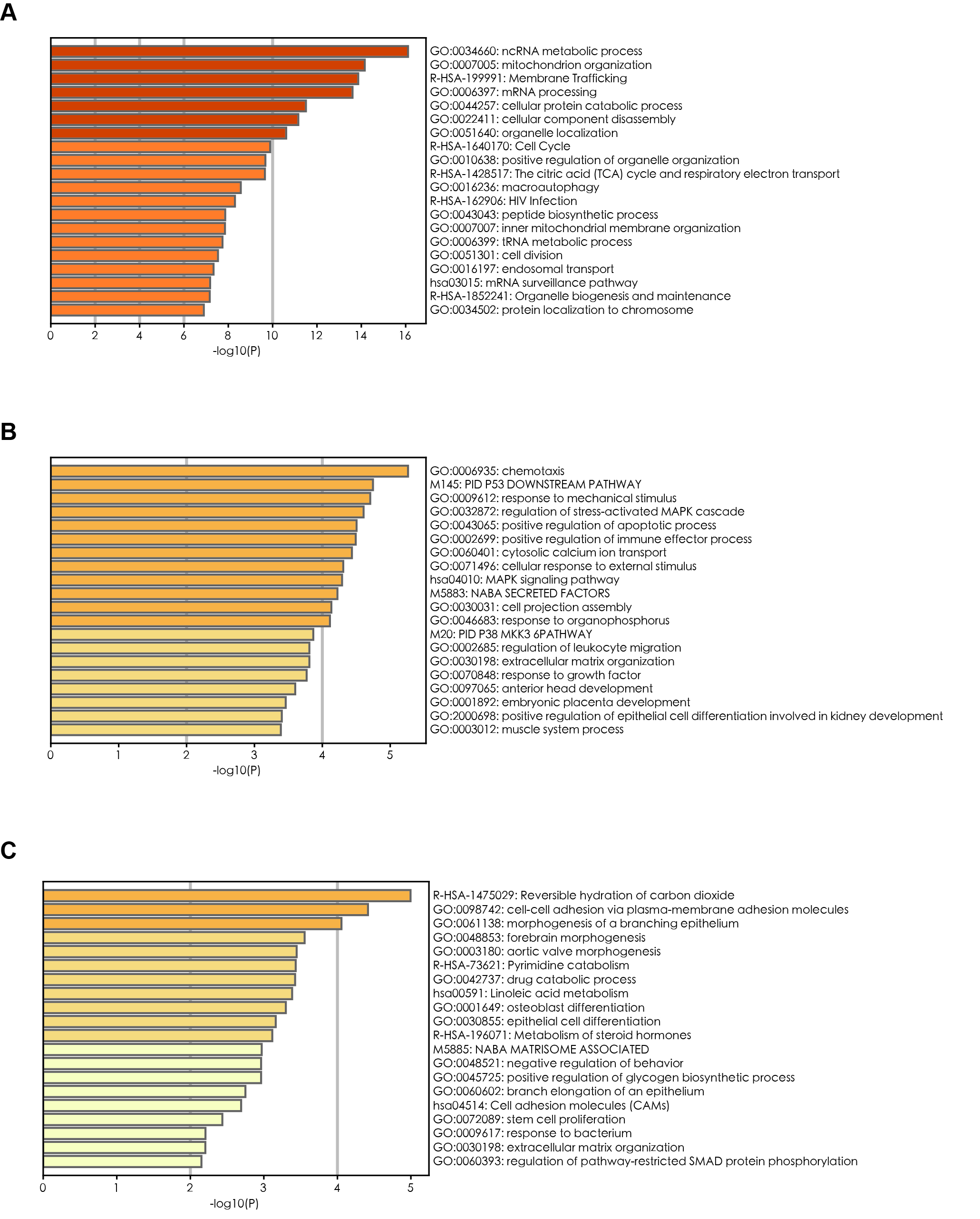

Supplement: FIGURE S1 — GO analysis of hub genes in different modules. (A) GO terms of hub genes in blue module. (B) GO terms of hub genes in yellow modules. (C) GO terms of hub genes in magenta module. [file Image_1.JPEG]
